# Red pepper drying with a double pass solar air heater integrated with aluminium cans

**DOI:** 10.1038/s41598-024-53563-6

**Published:** 2024-02-04

**Authors:** Zigale Admass, Ayodeji Olalekan Salau, Bimrew Mhari, Ewnetu Tefera

**Affiliations:** 1 Department of Mechanical Engineering, School of Mechanical and Chemical Engineering, Woldia University, Woldia, Ethiopia; 2 Department of Electrical/Electronics and Computer Engineering, Afe Babalola University, Ado-Ekiti, Nigeria; 3 School of Electrical and Computer Engineering, Debre Markos Institute of Technology, Debre Markos University, Debre Markos, Ethiopia; 4 School of Mechanical and Automotive Engineering, College of Engineering and Technology, Dilla University, P.O. Box 419, Dilla, Ethiopia; 5 Saveetha School of Engineering, Saveetha Institute of Medical and Technical Sciences, Chennai, Tamil Nadu India

**Keywords:** Engineering, Materials science

## Abstract

In this paper, an experimental evaluation of a newly developed flat plate double pass solar air heater combined with aluminum cans for drying red pepper was presented. The proposed solar dryer system was designed, modeled, and evaluated. Solar air heater trials were carried out using the absorber’s top and bottom plate and aluminum cans for red pepper drying at Bahir Dar, Ethiopia. To test the solar dryer, 100 pieces of red paper were obtained from the Bahir Dar region of Ethiopia for the purpose of experimentation. Microsoft Excel was used to perform statistical analysis of eleven mathematical models. The results show that the mixed-mode solar greenhouse dryer takes less time to dry red pepper than the open solar dryer. In the midday, the solar insolation reached 973 W/m^2^ and the minimum solar insolation was 220 W/m^2^ and air is expelled at a rate of 0.0383 kg/s. According to the experimental results, the dryers chamber temperature ranged from 30.9 to 54 °C, while the ambient temperature was between 22.6 and 28.2 °C. The mixed-mode double pass achieves up to 46% and 28% efficiency when used with aluminum can dryers and conventional open sun dryers, respectively. A drying rate of 0.0003395 kg/s was achieved for the open sun dryer system and 0.0000365 kg/s for the mixed mode solar dryer. Using mixed-mode and open-sun solar dryers, the logarithmic model was found to be most effective in explaining the red pepper behavior. Furthermore, a comparison was made between the experimental and predicted moisture ratios through the calculation of the coefficient of determination (R^2^), the reduced chi-square (X^2^), and the root mean square error (RMSE). The results show that the logarithmic model achieved the highest value of the correlation coefficient (R^2^), which was determined to be 0.9978 and 0.9989, while the logarithmic model achieved the lowest value of Chi-square (X^2^).

## Introduction

Solar drying is the oldest technological process for the preservation of food, crops, vegetables, and fruits by exposing agricultural products to the sun using solar energy. Solar heat causes the agricultural product to be dried, removing moisture (dewatering) to the desired value. Particularly, it is essential to reduce the moisture content of foods so that they can be stored for a long time^1,2^. As moisture content of foodstuffs is reduced, bacterial, yeast, and mold growth and reproduction are reduced, contributing to decay and minimising moisture-mediated deterioration. This method of preserving food ensures that it can be stored for a long period of time without spoiling^3^.

Drying techniques may be divided into four general categories based on the way of heated product open-air or natural dryers, solar drying takes place when the red pepper is exposed to the sun and wind by placing it in the filial area, on racks, or the ground. The sun directly dries red pepper by enclosing it in a clear container with a lid, allowing it to be directly exposed to the sunlight. Besides heating the air directly with solar radiation, the greenhouse effect traps heat inside the enclosure and raises its temperature. Unlike direct solar driers, indirect solar dryers heat fresh air in a separate collector from the food chamber. Direct sunlight damages foods' nutritional value, especially those that lose nutrients^4–6^.

During the warmer months, the greenhouse dryer works as a solar dryer, as well as protecting the product from rain, dust, insects, and animals. It is a simple structure, large load capacity, and relatively good thermal performance^7^. Direct solar greenhouse drying system (SGDS) requires only one greenhouse, while indirect solar greenhouse drying system consists of two main components: solar air collectors and greenhouses^8^. This study proposes a mixed-mode solar air collector consisting of a flat plate solar air collector with aluminium canes above and below the black painted absorber to increase the area of absorbing solar energy and to create turbulence.

The rest of the paper is structured as follows: Section “Related work” presents the related works. Section “Materials and methods” provides a detailed description of the proposed methodology. The experimental findings and analyses are provided in Section  “Results and discussion”. Finally, the paper is concluded in Section “Conclusion and recommendation for future work”.

## Related work

Authors in^9^ investigated a solar dryer fitted with a unique heat recovery technology, which was taken into account in the current study. The evaluation experiments were carried out at various flow rates and drying air relative humidity values.

In^10^, a phase change material (PCM) was introduced inside the rectangular aluminium tube utilized as the thermal absorber, and the performance of the cross-matrix absorber double-pass solar air heater (CMADPSAH) integrated with the PCM as thermal energy storage was investigated. Although, the authors didn’t mention the test materials used to evaluate the system.

The authors in^11^ examined the thermal performance of two counter flow double pass solar air heater (DPSAH) systems. In the first arrangement, a typical flat plate solar absorber was used, while in the second, water-filled tubular capsules were used as a sensible heat storage medium. The longitudinal installation of the tubular capsules is parallel to the airflow direction.

The authors in^12^ presented a newly developed Double-pass solar drier (DPSD) that was compared to a normal cabinet drier (CD) and a traditional open-air sun drying for drying red chilli in central Vietnam.

This paper presents a double pass solar air heater for a red pepper dryer which has a relatively better mass flow rate (MFR), average ambient temperature, solar radiation requirement, and size as compared to existing works.

## Materials and methods

### Dryer description

The dryer has an active mixed mode with aluminium cane collectors, a greenhouse to hold the product being dried, and direct solar energy transmission. The fan has to be used to facilitate air circulation. The sun's rays are captured through the transparent top cover on the solar collector and greenhouse, and the black-painted absorber plate coupled with aluminium cans is used to enhance this process. For the purpose of experimentation, 100 pieces of red paper were acquired from the Bahir Dar region in Ethiopia to test the solar dryer. Climate data and measured data are shown in Table 1, while the formulas and results of analytical calculations are shown in Table 2. The solar air collector is designed according to the dryer load of a greenhouse. To calculate the drying time, 80 kg of red pepper is dried from an initial moisture content of 79% on a wet basis to a final moisture content of 10–12.5% on a dry basis in 50 h^13,14^.

**Table 1:** Measurements, design parameters and calculations.

Items	Optimum values and assumption
Location	Bahir Dar (latitude 11.6 and longitude 38)
Crop	Red Pepper
Design capacity	80 kg
Bulk density	256 kg/m^3^
Maximum allowable temperature for red pepper	65 °C
Initial moisture content	79% (test)
Final moisture content	(10–12.5%)
Average pepper thickness	26 mm (Design)
Drying time per day	9 h (2:00 to 11:00)
Solar insolation	600.319 W/m^2^
Collector type	Double pass flat plate integrated with aluminum cans
Collector tilt angle	21.4°
Blowing rate	0.0383 m^3^/s (selection)
Density of air	${\rho }_{a}=1.2\mathrm{ kg}/{{\text{m}}}^{3}$
Specific heat capacity of the air	${C}_{pa}=1005\frac{{\text{J}}}{{\text{kg}}}.{\text{K}}$
Absorber thickness	1.25 mm
Air gap	10 mm
Glass thickness	4 mm
The vertical distance between adjacent trays	300 mm

**Table 2:** Formulas for analytical calculations and their values.

No	Name	Symbol	Formula	Value
1	Flat plate collector area	${A}_{c}$	${A}_{c}=\frac{Q}{I{\eta }_{d}{t}_{d}}$	$2{m}^{2}$
2	Mass of the moisture	$MW$	$\frac{{{\text{m}}}_{{\text{T}}}({{\text{W}}}_{{\text{o}}}-{{\text{W}}}_{{\text{f}}})}{100-{{\text{W}}}_{{\text{f}}}}$	$61.123Kg$
3	Amount of heat needed to evaporate moisture	$Q$	${M}_{i}{L}_{v}$	$105.85$ MJ (2943.42W)
4	Average solar insolation	${{\text{I}}}_{{\text{t}}}$		600.319 W/m^2^
5	Total volume of the drying chamber	${W}_{we}$	${W}_{we}={\rho }_{b}{V}_{T}$	0.3125 m^3^
6	Volume of red pepper per tray	${V}_{T}$	${V}_{T}=n{W}_{T}{L}_{T}{Y}_{l}$	0.026 m
7	Mass flow rate of air	${{\text{m}}}_{{\text{a}}}$	${\uprho }_{{\text{a}}}\times {{\text{V}}}_{{\text{a}}}$	$0.025 Kg/s$
8	Drying rate	${D}_{r}$	$\frac{dM}{dt}$	$3.395 x {10}^{-4} Kg/s$

**Figure 1:**
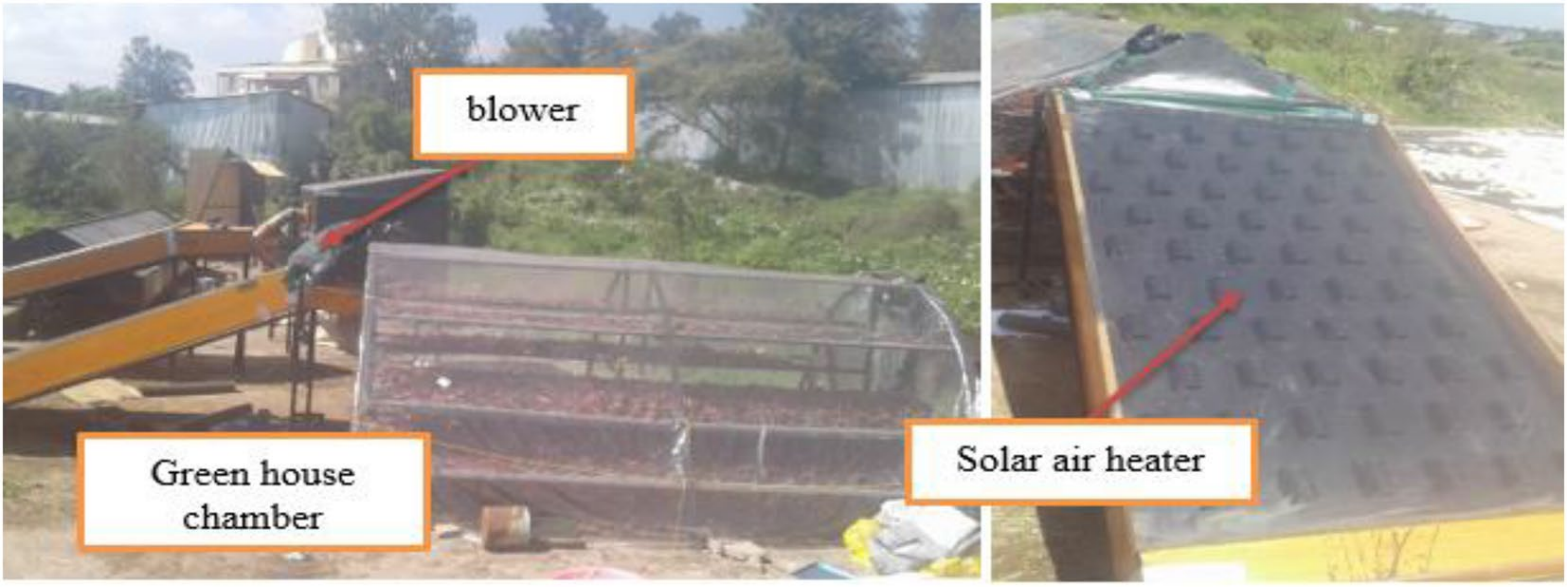
Mixed-mode solar red pepper dryer.

The manufactured flat plate solar air heater (SAH) and greenhouse drier was fashioned and installed on a basic iron frame positioned at 21.4° to the south, as determined by analytic calculations and shown in Fig. 1. There is a fan installed at the exit of the greenhouse dryer A blower unit, a test duct with entrance and exit sections, and various temperature measurement devices are included in the experimental setup. The flow system consists of an entry section, a test section, an exit section, a flow meter, and a centrifugal blower^13,15^. Double pass solar collector setup consists of a glazing glass single layer of 1 m × 2 m size by 4 mm thickness, a black paint aluminium absorber plate 1 m × 2 m by 1.25 mm thickness integrated with aluminium cans 100 mm length, 50 mm diameter, and 2 mm thickness. As part of the experiment, temperature, airspeed, solar radiation, relative humidity, the mass of the red pepper, and the moisture content of the red pepper are measured. From September to December, the mean temperature ranges between 22.6 and 28.2 °C, and the average relative humidity ranges from 89.47 to 59.95%. The red pepper was dried at 70 °C without compromising its quality, and the final moisture content of red pepper should be between 10 and 12.5% for safe storage.

### Mathematical modeling method

To select the best drying curve equation for describing the experimental data, it is important to evaluate its goodness of fit based on the deviation between the experimental values and predicted values for the mathematical models. There are two types of mathematical models: physics-based models, which begin with a physical law or model, and observation-based models, which start with experimental data^16^.

### Model validation

The initial mass of the samples of harvested red pepper and the final mass of dried red pepper were measured with the help of a weighing balance. The dried red pepper drying curves were analysed to find the most convenient moisture ratio (MR) model between the test and model values as shown in Table 3^15^. By comparing R^2^, RMSE, and X^2^, we selected the most suitable regression model.

**Table 3:** Drying kinetics descripting using mathematical models.

Model name	Model equation
Newton	$MR={\text{exp}}(-kt)$
Page	$MR=e{xp(-kt}^{n})$
Henderson and pabis	$MR=a{\text{exp}}(-kt)$
Logarithmic	$MR=a{\text{exp}}(-kt)+c$
Two terms	$MR=a{\text{exp}}(-{k}_{o}t)+b{\text{exp}}(-{k}_{1}t)$
Two-term exponentials	$MR=a{\text{exp}}(-kt)+(1-a){\text{exp}}(-kat)$
Wang and Singh	$MR=1+at+{bt}^{2}$
Diffusion approach	$MR=a{\text{exp}}(-kt)+(1-a){\text{exp}}(-kbt)$
Modified Henderson and Pabis	$MR=a{\text{exp}}\left(-kt\right)+b{\text{exp}}\left(-gt\right)+c{\text{exp}}(-ht)$

The maximum correlation coefficient is given by Eq. (1).

1 $${R}^{2}=1-\frac{{\sum }_{i=1}^{N}{\left({MR}_{pr,i}-{MR}_{ex,i}\right)}^{2}}{{\sum }_{i=1}^{N}{\left({MR}_{pr,mean}-{MR}_{ex,i}\right)}^{2}}$$

Minimum Chi-square is given by Eq. (2).

2 $${X}^{2}=\frac{{\sum }_{i=1}^{N}{({MR}_{ex,i}-{MR}_{pr,i})}^{2}}{N-n}$$

Minimum root means square error is given by Eq. (3).

3 $$RMSE=\sqrt{\frac{1}{N}{\sum }_{i=1}^{N}{({MR}_{pr,i}-{MR}_{ex,i})}^{2}}$$

The solar collector's steady-state thermal efficiency is given by Eq. (4).

4 $${\eta }_{c}=\frac{{\dot{m}}_{a}{C}_{pa}({T}_{c}-{T}_{a})}{{A}_{c}{I}_{T}}$$

The mathematical formula for expressing system efficiency is given by Eq. (5).

5 $${\eta }_{d}=\frac{{M}_{w}{L}_{v}}{{I}_{t}{A}_{c}t}$$

The experiments is conducted according to the thin-layer drying concept given by Eq. (6)

6 $${D}_{r}=\frac{dM}{dt}$$

The moisture content of the red pepper is calculated using Eq. (7).

7 $$\left(MC \%\right)=\frac{{M}_{i}-{M}_{f}}{{M}_{i}}x100\%$$

The authors assumed a 40% impeller efficiency to cater to most fans since cost and power are major considerations as the fan efficiency ranges between 30 and 70%. Authors affirm that the study complies with relevant institutional, national, and international guidelines and legislation for plant ethics.

## Results and discussion

**Figure 2:**
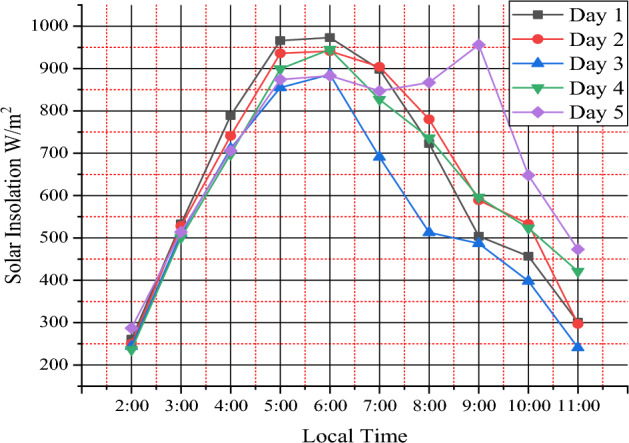
Variation of solar intensity with time for five days.

**Figure 3:**
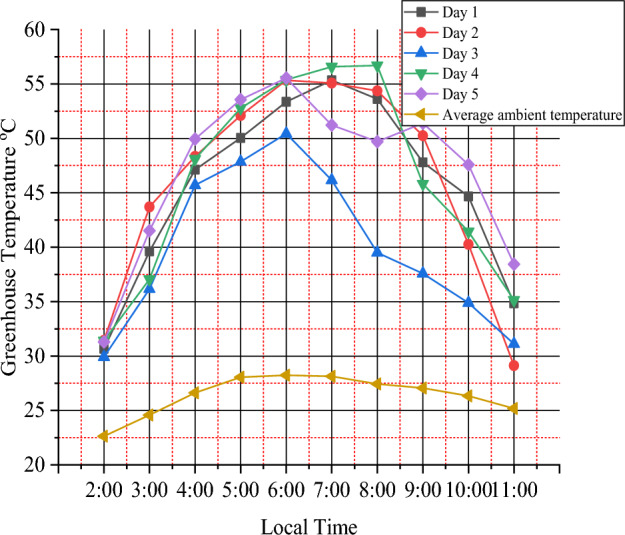
Average temperatures in the greenhouse drying chamber vs Local time.

The experiment was conducted between 2:00 and 11:00 Ethiopian local time which gets optimum day temperature between these times. The highest temperature in the greenhouse chamber was recorded to be from 30.9 to 54 °C over ambient temperature of 22.6 °C to 28.2 °C and average relative humidity from 89.47 to 59.94%. The average temperature rises over ambient during the study observed to be 18.5 °C. Solar insolation is plotted as a function of time of day in Fig. 2. The maximum value of solar insolation recorded was 973W/m^2^ on the first day at 6:00 h local time and a minimum of 236 W/m^2^ on the fourth day at 2:00 local time. Figure 3 shows maximum greenhouse temperatures were 56.7 °C at 8:00 local time on day four. This shows that the mixed-mode solar dryer has better performance than the open-air sun drying system because the temperature in the greenhouse is much more than the ambient temperature.

### Variation of temperature vs local time

**Figure 4:**
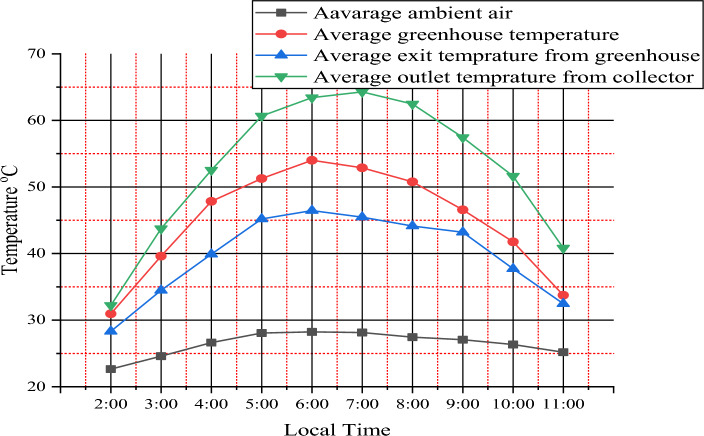
Variation temperatures versus local time.

During most daylight hours, the temperature inside the dryer and solar collector was much higher than the ambient temperature. Figure 4 shows the temperature variations in the ambient air, greenhouse drying chamber, greenhouse exit, and solar collector outlet**.**

### Performance evaluation of the pepper dryer

The greenhouse drying system was evaluated based on its solar air collector efficiency, drying rate of red peppers, moisture loss, and drying efficiency. Efficiencies of double pass flat plate solar air heaters are the ratio of the heat gained by the air leaving the collector to the incident solar energy^17^. The solar collector's steady-state thermal efficiency is given by Eq. (4). The bio chemical quality parameters for the red pepper are discussed in^17^.

The efficiency of the double pass flat plate solar air heater was calculated using average solar collector outlet temperature, ambient temperature, and solar insolation data collected within a given time interval using MFR of 0.025 kg/s as shown in Table 4.

**Table 4:** Double pass flat plate solar air heater efficiency.

Time	Collector outlet temperature	Ambient temperature	Solar insolation	The efficiency of double pass solar collector (%)
2:00	32.22	22.64	255.6	0.72
3:00	43.74	24.58	517	0.71
4:00	52.54	26.62	729	0.68
5:00	60.66	28.06	906	0.69
6:00	63.42	28.24	925.6	0.73
7:00	64.28	28.14	833.4	0.83
8:00	62.46	27.44	723.8	0.93
9:00	57.44	27.06	626.4	0.93
10:00	51.62	26.34	511.8	0.95
11:00	40.82	25.18	346.6	0.86
Average	52.92	26.43	637.52	0.79

The efficiency of the collector achieves the highest value in the afternoon. This is caused by high gains of solar energy from the sun due to a ratio of beam radiation on the tilted surface to that of the horizontal surface. The efficiency is increasing from morning time and decrease after 10:00 h. Unfortunately, in the morning time almost constant efficiency results due to weather conditions at testing time. The overall result of the double pass solar collector average efficiency was recorded as 79% which is much greater than the previous flat plate solar heater's efficiency. Making the flow of air a double pass and adding aluminium can roughness in the absorber optimizes the efficiency of the heater.

### Mixed-mode and open sun drying system efficiency

The mathematical formula for expressing system efficiency is given by Eq. (5) ^18^. Open-sun drying efficiency is shown in Table 5 and mixed-mode solar drying efficiency is shown in Table 6. The drying efficiency is calculated using temperature variations. The drying efficiency decreases when the drying time is increases due to reduction in the moisture level of red pepper in both drying systems. The drying efficiency was seen to be high on the first day compared to the following days in both open sun drying and mixed-mode greenhouse drying systems, with an average drying efficiency of 28% and 46% recorded at average product temperatures of 26.23 °C and 44.93 °C respectively.

**Table 5:** Open sun drying system efficiency.

Day	Ambient temperature (T_pr_, °C)	Mass of water removed (M_w_, kg)	Latent heat of pepper at T_pr_ (L_v_, kJ/kg)	Solar insolation (I_t_, W/m^2^)	Efficiency (η_d_)
1	27.53	12.8	1794.552	640.5	0.49
2	26.7	10.9	1796.497	649.9	0.42
3	24.89	9.2	1800.74	553.5	0.41
4	26.63	7.9	1796.661	638.2	0.31
5	26.4	6.8	1797.2	705.5	0.24
6	26.08	5.9	1797.951	657.4	0.22
7	25.67	5.1	1798.912	659	0.19
8	25.85	4.2	1798.49	657.5	0.16
9	26.31	3.5	1797.411	697.4	0.13
Average	26.23	7.37	1797.6	650.99	0.28

**Table 6:** Mixed-mode solar drying system efficiency.

Days	Greenhouse temperature (T_pr_, °C)	Mass of water removed (M_w_, kg)	Latent heat of pepper at T_pr_ (L_v_, kJ/kg)	Solar insolation (I_t_, W/m^2^)	Efficiency (η_d_)
1	45.7	21.5	1751.958	640.5	0.82
2	46	15.4	1751.255	649.9	0.57
3	39.93	10.9	1765.484	553.5	0.48
4	46.04	7.3	1751.161	638.2	0.28
5	47.02	5	1748.864	705.5	0.17
Average	44.938	12.02	1753.744	637.52	0.46

### Average drying time and drying rate

**Figure 5:**
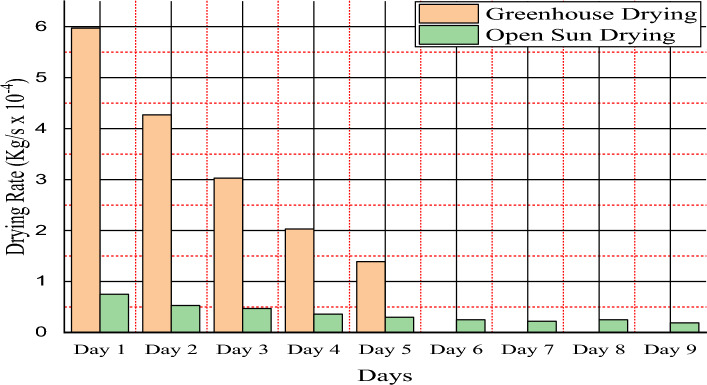
Drying rate variation between mixed-mode and open-mode solar systems.

According to the difference in moisture content, the drying rate is the amount of moisture removed from the red pepper per unit time. The experiments were conducted according to the thin-layer drying concept given by Eq. (6) ^19^. The drying rate was calculated using a mass of water removed at a specified time. Using the mixed-mode greenhouse solar dryer, 61.123 kg of moisture was removed at a drying time of 50 h and in the open sun solar dryer, 11.84 kg of moisture was removed at a drying time of 90 h from 80 and 15.5 kg mass of fresh harvested red pepper. Mixed-mode and open-sun solar dryer systems have drying rates of 0.0003395 kg/s and 0.0000365 kg/s, respectively. Figure 5 illustrates the difference between solar drying rates obtained using the mixed-mode and open-mode systems. In the mixed mode, the drying rate was observed high value compare to the open sun drying system. This shows that the moisture removal rate of red pepper of the mixed-mode drying system is faster than that of the open sun drying.

### Percentage moisture of red peppers

Equation (7) is used to calculate the moisture content of the product when it is dry^20^. The experiment was measured by an oven dryer taking three samples. The moisture content of freshly harvested red pepper is shown in Table 7 at their initial moisture content. Fresh harvested red peppers has an average moisture content of 79%.

**Table 7:** Moisture content of freshly harvested red pepper.

Sample	Initial weight in (g)	Weight after 24 h (g)	Weight after 26 h (g)	Moisture content
1	68	17	17	75%
2	63	13	13	79%
3	55	9	9	83%
				Average = 79%

**Figure 6:**
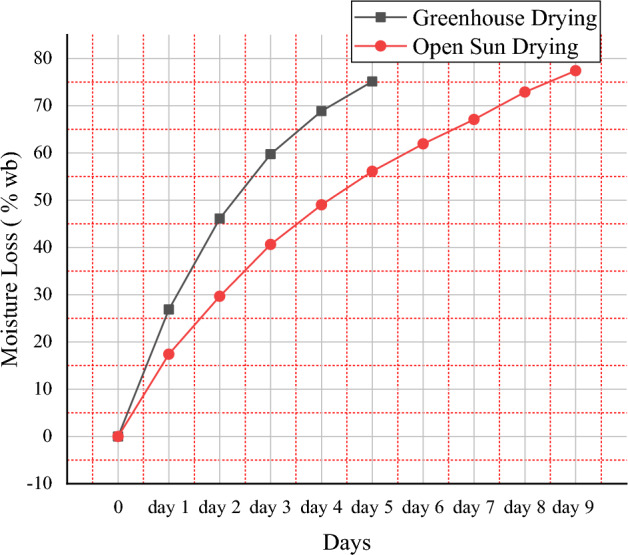
Comparison of moisture loss of mixed-mode solar drying versus open sun drying.

**Figure 7:**
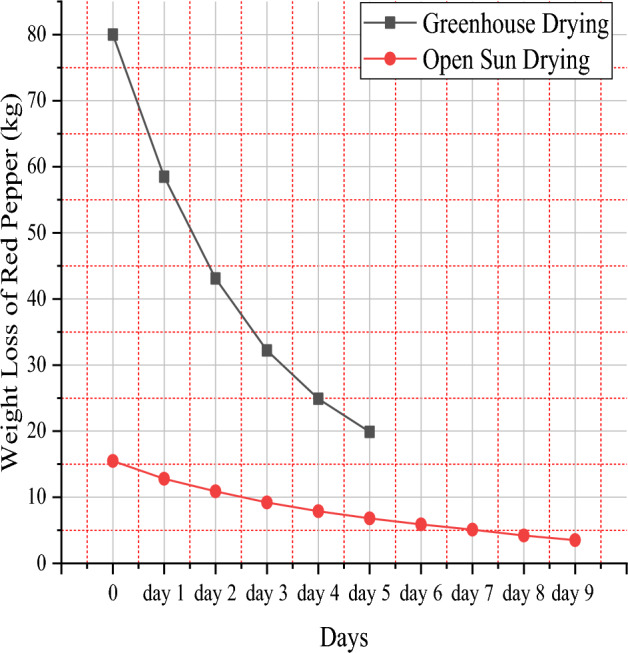
Weight loss of red pepper under open sun versus greenhouse drying.

Figure 6 depicts an examination of moisture loss (%wb) for the sample retrieved from the mixed-mode dryer and the sun dryer comparison. Red pepper requires 50 h to remove 75.13% (wb) of moisture content using the greenhouse drier method, and 90 h to remove 77.42% (wb) of moisture content using the open sun method. The moisture loss under the mixed-mode greenhouse dryer is faster than the standard open sun drying system to attain the target moisture level of 11.5% (DB). To achieve the necessary moisture level, the mixed-mode greenhouse solar drying system took five days, while open sun drying took nine days. This experiment was carried out at the start and end of the day for a period of 9 days, as shown in Fig. 7.

80 kg of fresh red peppers were used as the beginning weight for the mixed-mode solar dryer, and after five days, 19.9 kg of red peppers and 15.5 kg from the open sun dryer system were recorded, with the final mass of pepper being recorded at 3.5 kg to achieve the necessary moisture level.

**Figure 8:**
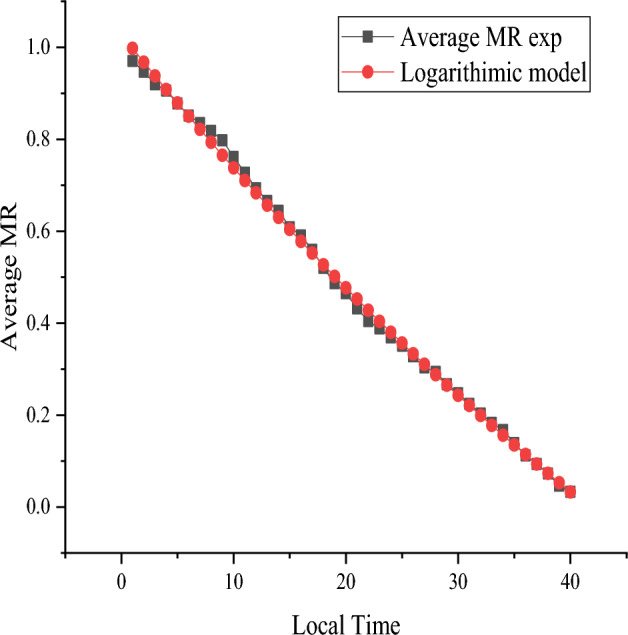
Logarithmic model for mixed-mode solar drying.

**Figure 9:**
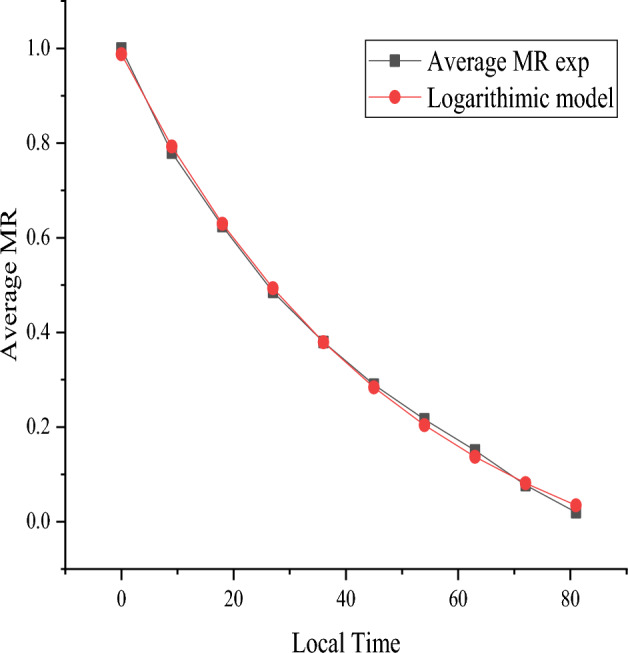
Logarithmic model for open sun drying.

As observed in Figs. 8 and 9, the experimental MR values were fitted using all eleven of the models from the OriginPro 2019b software in both the mixed-mode solar drying mode and the open sun drying mode. The lowest value of Chi-square X^2^ was found to be 0.00019 and 0.00014, while the logarithmic model displayed the greatest value of the correlation coefficient R^2^, which was determined to be 0.9978 and 0.9989. In addition, across all the mathematical models, regardless of average tray placements, the lowest root mean square error (RMSE) was 0.013265 and 0.010035, respectively.

The model coefficients and constants regression analysis of natural logarithm of MR on drying time for all mixed-mode greenhouse and open-air drying systems were determined using Microsoft Excel, as shown in Tables 8 and 9, as well as the goodness of fit values for R^2^, X^2^, and RMSE. The values were found to fluctuate with temperature^21–32^.

**Table 8:** Estimated values of parameters of different applied mathematical models for mixed-mode drying system.

Model name	Constants and coefficients	R^2^	${x}^{2}$	RMSE
Newton	k = 0.041755	0.971319	0.005837	0.075437
Page	k = 0.007388; n = 1.557322	0.993331	0.000636	0.02459
Henderson and pabis	a = 1.137841; k = 0.048501	0.959801	0.003717	0.059424
**Logarithmic**	**a = 2.828839; k = 0.010843; c = -1.80046**	**0.99785**	**0.00019**	**0.013265**
Two term	a = 0.568922; k_0_ = 0.048501; b = 0.568922; k_1_ = 0.048501	0.959801	0.003924	0.059424
Two term exponentials	a = 0.995162; k = 0.041757	0.971318	0.00599	0.075437
Wang and Singh	a = − 0.0273; b = 0.0000722	0.997551	0.000238	0.015025
Diffusion approach	a = − 4.25342; k = 0.101609; b = 0.814744	0.988902	0.001028	0.03083
Modified Henderson and Pabis	a = 0.379281; k = 0.048501; b = 0.379281; g = 0.048501; c = 0.379281; h = 0.048501	0.959801	0.003924	0.059424

Significant values are in bold.

**Table 9:** Estimated values of parameters of different applied mathematical models for open sun drying system.

Model name	Coefficient and constants	R^2^	${x}^{2}$	RMSE
Newton	k = 0.028723	0.991156	0.00121	0.033003
Page	k = 0.016886; n = 1.144608	0.993561	0.000771	0.024843
Henderson and pabis	a = 1.02049; k = 0.029324	0.990121	0.001281	0.032018
**Logarithmic**	**a = 1.191759; k = 0.019862; c = − 0.204**	**0.998904**	**0.000144**	**0.010035**
Two term	a = 0.510246; k_0_ = 0.029324; b = 0.510246; k_1_ = 0.029324	0.99012	0.001709	0.032018
Two term exponentials	a = 0.008594; k = 3.307687	0.991029	0.001434	0.033871
Wang and Singh	a = − 0.0215; b = 0.00012	0.995794	0.000625	0.022359
Diffusion approach	a = − 1.61301; k = 0.051483; b = 0.781511	0.994094	0.000808	0.023779
Modified Henderson and Pabis	a = 0.340164; k = 0.029324; b = 0.340164; g = 0.029324; c = 0.340164; h = 0.029324	0.99012	0.002563	0.032018

Significant values are in bold.

For both mixed-mode solar greenhouses and sun drying of red pepper, the logarithmic model was deemed the most suitable thin layer model. On the basis of the Figures, the curves show a good correlation between the experimental and computed values of MR, where the values are distributed around a straight line. This confirms the selection of models in describing the drying behaviour of red pepper.

### Solar dryer performance

Table 10 presents a comparison of the performance of the proposed solar dryer system to existing systems. In comparison with previous works, the findings of this study indicate that the suggested DPSAH for drying red peppers has a better mass flow rate, improvement in the average ambient temperature, and an improved solar radiation capacity. The results show that the proposed system achieved a higher drying capability than other systems as shown by and minimizes moisture-mediated deterioration.

**Table 10:** Comparison of proposed solar dryer system with existing systems.

Author	Item to be dried	Mass flow rate (kg/s)	Amount of heat needed to evaporate moisture (W)	Average relative humidity ranges/ambient temperature	Solar insolation/radiation	Drying time per day (h)
^9^	Onion	0.016	700	21–35 °C	1010 W/m^2^	5
^10^	Agricultural produce	0.004	127	–/53 °C	1017 W/m^2^ and 944 W/m^2^ for phase 1 and phase 2	10
^11^	–	0.03	–	–/25 °C	1000 W/m^2^	–
^12^	Red chilli	–	–	Ranges from 5 and 95%/	22.30 W/m^2^	73
^13^	Red pepper	0.047	1004	Ranges from 17.6% to 62.6%/40 °C	0–1000 W/m^2^	7
Proposed	Red pepper	0.025	2943.42	Ranges from 89.47% to 59.95%/22.6 °C and 28.2 °C	973 W/m^2^	9

## Conclusion and recommendation for future work

### Conclusion

This paper presents the experimental evaluation of a newly designed flat plate double pass solar air heater integrated with aluminium can for red pepper drying. The following conclusions were drawn from this study:

- It was observed that greenhouses achieves the optimal temperature than separate flat plate dryers and greenhouse dryers.
- With the newly designed system, the maximum outlet temperature is 54 °C for an ambient temperature of 28.2 °C.
- A batch of red pepper weighing 80 kg and 15.5 kg by mass, whose initial moisture content is 79% wet basis, can be dried to 11% moisture content using the proposed double pass mixed mode solar dryer and an open solar dryer system. The moisture loss rate, the drying rate, the collector efficiency, and the drying efficiency were used to evaluate the performance of the drying systems. The total dying duration of red pepper using the mixed mode greenhouse dryer system takes 50 h to remove 75.13% of moisture content and 90 h using the open sun drying technique to remove 77.42% moisture. Open sun drying yielded 0.000395 kg/s and mixed-mode greenhouse solar drying yielded 0.0000365 kg/s, respectively.
- In order to select the best drying model, the R^2^, X^2^, and RMSE values were compared. A logarithmic model is appropriate for both mixed-mode and open sun drying. Solar dryers designed with aluminum cans in the absorber's top and bottom have values of R^2^, X^2^, and RMSE of 0.99, 0.00019, and 0.0132, respectively. The results of the regression shows that the newly designed system has no effect on the food quality of the red pepper.

### Recommendation for future work

In the future, authors hope to use deep learning methods to determine the best drying models.

## Data Availability

The datasets generated during and/or analysed during the current study are not publicly available but are available from the corresponding author on reasonable request.

## Abbreviations

CD: Cabinet drier
CMADPSAH: Cross-matrix absorber double-pass solar air heater
DPSAH: Double pass solar air heater
MFR: Mass flow rate
MR: Moisture ratio
PCM: Phase change material
RMSE: Root means square error
R^2^: Correlation coefficient
SAH: Solar air heater
X^2^: Chi-square
